# Repair of a chondral defect using a cell free scaffold in a young patient - a case report of successful scaffold transformation and colonisation

**DOI:** 10.1186/1471-2482-13-11

**Published:** 2013-04-16

**Authors:** Karl F Schüettler, Johannes Struewer, Marga B Rominger, Peter Rexin, Turgay Efe

**Affiliations:** 1 Department of Orthopedics and Rheumatology, University Hospital Marburg, Baldingerstrasse, Marburg, 35043, Germany; 2 Department of Radiology, University Hospital Marburg, Baldingerstrasse, Marburg, 35043, Germany; 3 Institute of Pathology, University Hospital Marburg, Baldingerstrasse, Marburg, 35043, Germany

**Keywords:** Vital chondrocyte, Cell-free scaffold, Histological examination, Chondrocyte migration

## Abstract

**Background:**

Chondral defects of the articular surface are a common condition that can lead to osteoarthritis if not treated. Therapy of this condition is a topic of constant debate and a variety of chondral repair strategies are currently used. One strategy involves implantation of a cell-free matrix of type I collagen (COL1), to provide a scaffold for chondrocyte migration and proliferation and extracellular matrix production. Although several studies have suggested that chondrocytes can move, to the best of our knowledge there is still no proof of chondrocyte occurrence in a former cell-free scaffold for articular cartilage repair in humans.

**Case presentation:**

An 18-year-old male patient underwent arthroscopic surgery of the knee for patellar instability and a chondral defect of the femoral condyle. Clinical outcome scores were recorded pre-operatively, after 6 weeks and after 6, 12, 24 and 36 months. MRI was recorded after 6 weeks and after 6, 12, 24 and 36 months postoperatively. At 42 months after implantation of a cell-free type I collagen matrix and reconstruction of the medial patellofemoral ligament, the patient was again treated arthroscopically for a tear of the medial meniscus of the same knee. A biopsy of the previous chondral defect was taken during arthroscopy for histological examination.

**Conclusion:**

In addition to good clinical and radiological results reported for cell-free scaffolds for cartilage repair in several other studies, transformation of the scaffold could be observed during re-arthroscopy for the meniscal tear. Histological examination of the specimen revealed articular cartilage with vital chondrocytes and a strong staining reaction for type II collagen (COL II), but no reaction for type I collagen staining. This might indicate a complete transformation of the scaffold and supports the theory that cell free scaffolds could support cell migration. Although the cell source remains unclear, migrating chondrocytes from the periphery remain a possibility.

## Background

Articular surface chondral and osteochondral lesions are a common problem, affecting patients in all age groups. Curl et al. assessed the patient population that might benefit from cartilage grafting by reviewing 31,516 knee arthroscopies performed between June 1991 and October 1995 [1]. Cartilage lesions were documented for 19,827 patients (63%). Lesions caused by traumatic injuries, degenerative joint disease or osteochondritis dissecans led to osteoarthritis of the affected joint [2,3].

Several different treatments are available for small articular chondral defects, such as osteochondral transfer and bone marrow stimulation [4-6]. However, repair tissue after bone marrow stimulation has limited biochemical and biomechanical properties, and donor site morbidity has an adverse effect on the outcome of osteochondral transfer [7,8]. Brittberg et al. were the first to describe autologous chondrocyte implantation as a new treatment for articular chondral defects [9]. Over time, this method has evolved resulting in matrix-induced autologous chondrocyte growth on 3D scaffolds [10,11]. Although these techniques show promising results, they still have some limitations, such as high costs for the cell culture required, a time-consuming two-step procedure and donor site morbidity due to chondrocyte harvesting.

The next evolutionary step has been the use of cell-free scaffolds [12]. The basic principle of this procedure is the use of a cell-free type I collagen (COL1) matrix to fill the cartilage defect with a suitable matrix for chondrocyte migration and proliferation and production of extracellular matrix. Further advantages of these cell-free matrices are the one-step surgical procedure and avoidance of defects from cell harvesting. This technique shows promising clinical results [13].

Although possible mechanisms underlying chondrocyte mobility have been discussed [14] and promising data from animal models are available [12,15], proof of chondrocyte ingrowth and matrix transformation is still lacking for human patients.

## Case presentation

In August 2008, an 18-year-old male was admitted to our department for recurrent dislocation of the patella. Physical examination revealed a free range of motion (ROM; extension/flexion 0–0–130°) and a stable femoral–tibial joint with negative Lachmann and pivot shift tests. Diagnostic management included plain radiographs showing lateralisation of the patella. Magnetic resonance images (MRI) showed rupture of the medial patellofemoral ligament (MPFL) and a chondral lesion of the femoral condyle. The patient had suffered four re-dislocations, the last one without spontaneous reduction, so we performed MPFL reconstruction according to Schottle et al. [16] using the ipsilateral gracilis tendon and implanted a cell-free COL1 matrix (CaReS-1S®, Arthro-Kinetics, Krems, Austria) for treatment of the chondral lesion. The scaffold represents a three-dimensional collagen gel consisting of 4.8 mg/mL type I collagen derived from rat tails. The diameter was 15 mm and the thickness was 6 mm. The matrix was stored in sterile phosphate-buffered saline solution and preserved at 4°C until use.

**Figure 1:**
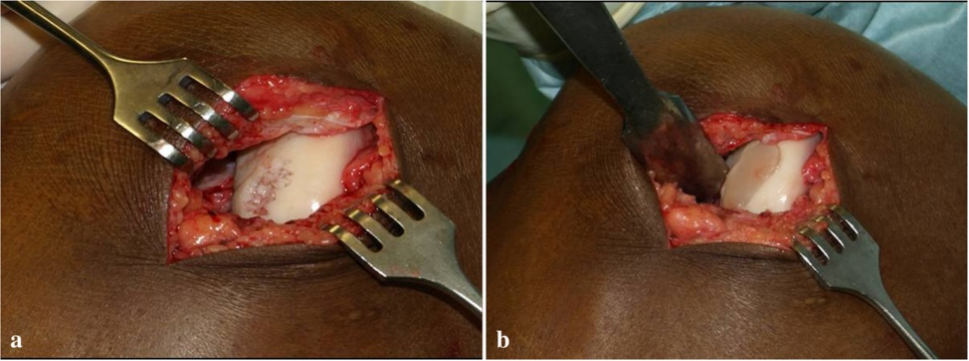
Intraoperative situs of the scaffold implantation.

The cell-free scaffold was implanted via a medial mini-arthrotomy. The chondral defect was carefully prepared using a cutter and a sharp angulated curette; the underlying subchondral bone was intact and without additional bleeding into the defect. The scaffold was implanted in a press-fit manner and showed complete congruity with the surrounding cartilage surface (Figure 1). The arthrotomy was closed layer-wise in a standard procedure.

The postoperative rehabilitation program started with immobilisation of the knee joint for 2 days. Then the patient was mobilised using two crutches with toe-touch weight bearing and limitation of flexion to 30° for the next 2 weeks (ROM extension/flexion 0–0–30°). From 3 to 6 weeks after surgery, flexion was allowed up to 90° and consecutively full weight bearing was achieved. Full ROM was allowed 6 weeks after surgery.

Follow-up was scheduled for 6 weeks and 6, 12, 24 and 36 months after surgery. MRI and functional, clinical and subjective assessment using the visual analogue scale (VAS [17]), the International Knee Documentation Committee scale (IKDC [18]) and the Tegner-Lysholm activity scale (Tegner [19]) were performed. MR images were scored according to the Magnetic Observation of Cartilage Repair Tissue (MOCART) scale (Table 1) [20,21].

**Table 1:** MOCART scores over time (w = weeks, m = months)

**Item**	**Scoring**	**Follow-up**				
		**6 w**	**6 m**	**12 m**	**24 m**	**36 m**
**Defect filling**	**Complete = 20**	**20**	**20**	**20**	**20**	**20**
	Hypertrophy = 15					
	Filling >50% = 10					
	Filling <50% = 5					
	Bone exposed = 0					
Integration with border zone	Complete = 15	10	10	15	15	15
	Split = 10					
	Integration >50% = 5					
	Integration <50% = 0					
Surface of repair tissue	Intact = 10	10	10	10	10	10
	>50% = 5					
	<50% = 0					
Structure of repair tissue	Homogeneous = 5	0	0	0	5	5
	Nonhomogeneous = 0					
Signal intensity for repair tissue	Normal = 30	0	0	15	15	15
	Nearly normal = 15					
Subchondral lamina	Abnormal = 0	5	5	5	5	5
	Intact = 5					
	Non-intact = 0					
Subchondral bone	Intact = 5	5	5	5	5	5
	Non-intact = 0					
Adhesions	No = 5	5	5	5	5	5
	Yes = 0					
Effusion	No = 5	0	5	5	5	5
	Yes = 0					
**Final score**		**55**	**60**	**80**	**85**	**85**

All MR images were obtained with a 1.5-Tesla MRI Scanner MAGNETOM Espree (Siemens, Erlangen,Germany). A knee coil with a field of view of 18 cm was used with the knee positioned in extension. The following standardised sequences were recorded for coronal, sagittal and transverse slice orientations: proton density turbospin-echo fast suppression (320 9 320; thickness 3 mm; repeat time (TR) 3,000 ms; echo time (TE) 37 ms); T1 (384 9 384; thickness 3 mm; TR 411 ms; TE 13 ms); T1-volume-interpolated breathhold examination (280 9 320; thickness 1.5 mm, TR 16; TE 7); and T2 (512 9 512; thickness 3 mm; TR 460 ms; TE 15 ms).

**Figure 2:**
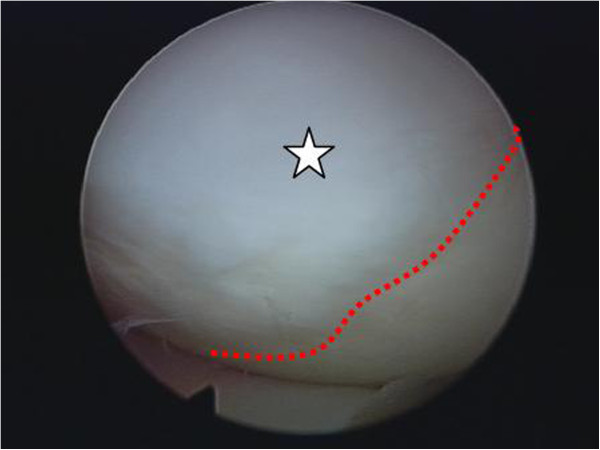
Intraoperative view of the scaffold 42 months after implantation (white banner).

In 2012 the same patient, now 22 years old, underwent arthroscopic surgery of the same knee, this time for a medial meniscal tear. Preoperative MRI showed a displaced medial meniscal tear. Before surgery, the patient gave informed consent for biopsy from the grafted chondral defect. The meniscal tear was repaired using arthroscopic sutures in all-inside technique with FasT-Fix (Smith&Nephew, Marl, Germany). The previous chondral defect showed good implant integration, with no signs of inflammation or dislocation (Figure 2) and a biopsy specimen was taken for histological examination using a spinal needle. The patient recovered quickly after arthroscopic surgery. Rehabilitation included partial weight-bearing exercise and limitation of flexion to 60° for 3 weeks. Full weight-bearing and a full ROM were achieved in week 6 after surgery.

### Histological preparation

Directly after surgery the specimen was fixed, decalcified and embedded using a standard procedure (24 h in 4% formalin, rinsed for 2 h with sterile water, decalcification in EDTA for 12 h, dehydration in increasing ethanol concentrations and standard paraffin embedding).

Slices of 3 to 4 μm were taken and stained after clearing the paraffin and rehydration. A CaReS-1S® cell-free scaffold was treated in exactly the same way for comparison. We first performed standard haematoxylin and eosin (H&E) staining, then immunhistochemical staining for COL1 and COL2. The primary COL1 antibody was a goat polyclonal antibody against COL1A1 (D-13, sc-25974, Santa Cruz Biotechnology, Santa Cruz, CA, USA; 1:50). The primary COL2 antibody was a mouse monoclonal antibody against COL2 clone 6B3 (MAB8887, Chemicon International, 1:200). The secondary COL1 antibody was a biotinylated anti-goat IgG antibody. For COL2 we used a peroxidase-labelled polyclonal antibody (HRP rabbit/mouse, Ref. V5007, Dako Envison Kit) with 3,3′-diaminobenzidine as chromogen.

### Results

**Figure 3:**
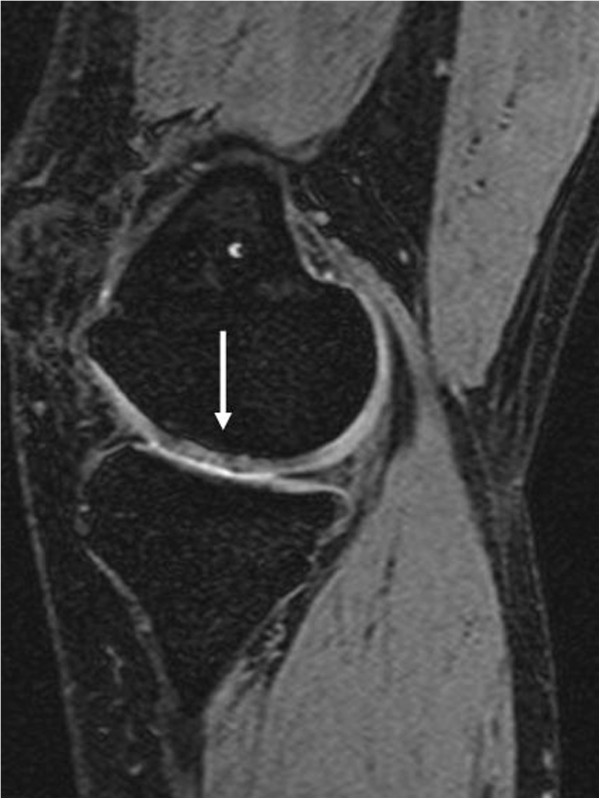
Follow-up MRI taken 36 months after surgery showing the cell-free scaffold (white arrow).

The clinical results were favourable and an improvement in function and a decrease in pain were evident from preoperative values (Table 2). Representative MRI image at 36 months postoperatively is shown in Figure 3. Using the MOCART scoring system there was a constant improvement from preoperative to 36 months postoperatively, indicating that the implant was correctly positioned and intact (Table 1).

**Table 2:** Changes in clinical scores over time (w = weeks, m = months)

**Score**	**Preoperative**	**Follow-up**				
		**6 w**	**6 m**	**12 m**	**24 m**	**36 m**
IKDC	66	84	100	90	90	91
VAS	2	0	0	2	1	1
Tegner	3	2	4	2	2	2

**Figure 4:**
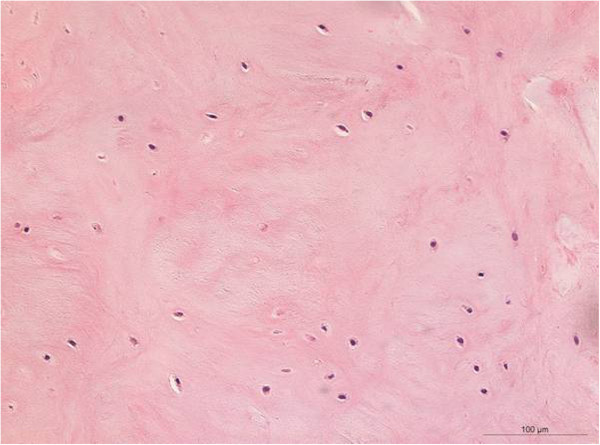
**H&E staining of regenerated cartilage showing vital chondrocytes and no signs of inflammation or abnormal calcification (magnification 200**×**).**

**Figure 5:**
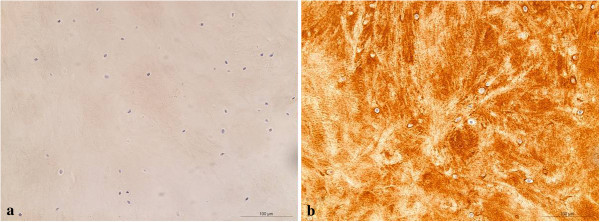
Regenerated cartilage showing no reaction for type I collagen (a) and a strong staining reaction for cartilage II collagen (b).

**Figure 6:**
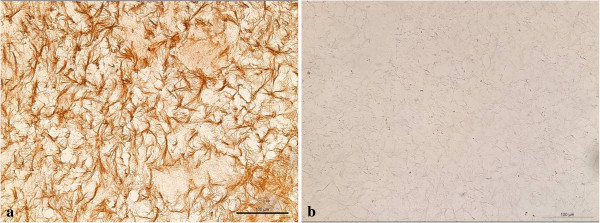
Scaffold showing a strong staining reaction for cartilage I collagen (a) and showing no reaction for type II collagen (b).

H&E staining showed nests of vital mononuclear isomorphic chondrocytes (Figure 4) within the regeneration tissue. There were no signs of inflammation, abnormal calcification or scar tissue. Immunohistological staining of the specimen showed a strong localisation for COL2 and no localisation for COL1 (Figures 5a and b), thus showing no trace of the former scaffold-matrix. In comparison, the bare scaffold stained as a negative control showed a strong staining reaction for COL1 and no reaction for COL2 (Figures 6a and b).

In summary, the specimen showed the typical appearance of articular cartilage, with no signs of remaining scaffold material, no abnormal calcification within the regeneration tissue and no signs of scar tissue.

### Discussion

The most important finding in the present case was that in addition to favourable clinical, functional and radiographic results, transformation of the cell-free COL1 scaffold took place. The biopsy showed no signs of remaining COL1, but instead showed COL2 with embedded vital chondrocytes.

Several studies have shown that the use of cell-free scaffolds leads to favourable results comparable to those for cell-seeded scaffolds in different animal models [10,12]. Similar results regarding clinical and morphologic outcome after implantation of cell-free COL1 matrices in humans were recently published [13].

The clinical course for the present case is in line with these results. After implantation of the cell-free scaffold, clinical, functional and morphological assessment revealed continuing improvement over time. The deterioration in results at the latest follow-up can undoubtedly be attributed to the traumatic meniscal tear the patient suffered. This injury also explains the slight deterioration in MOCART score due to the accompanying effusion, which is not associated with the implanted scaffold itself.

Histological and immunohistological results showed vital chondrocytes and a complete transformation of the cell-free COL1 matrix into a COL2 matrix. The cell-free COL1 scaffold thus facilitated the formation of good quality repair tissue - as confirmed by clinical, functional and morphologic assessment - with the histological appearance of articular cartilage.

Some open questions nonetheless still remain. Where did the chondrocytes that were found in the specimen come from? If they migrated from the perilesional tissue, how does chondrocyte migration occur and what cellular mechanisms are involved?

Morales reviewed the literature on chondrocyte movement in 2007 [14]. Although data on *in vitro* and *in vivo* examinations of chondrocyte motility were available at that time, the author concluded that *in vivo* chondrocyte motility remained to be proven.

Since the underlying bone was not penetrated, ingrowth from this direction seems rather unlikely but can not be excluded. Migration into the scaffold from the surrounding articular cartilage seems more likely; Lyman et al. [22] recently observed chondrocytes lining the margins of purposely created defects in an *ex vivo* organ model of human articular cartilage after 3–4 weeks in culture. Another possible origin of these cells could be traced to the presence of chondroprogenitor cells, which are also localised in the surrounding cartilage [23,24]. A further potential origin of these cells might be the synovial fluid which contains mesenchymal progenitor cells that could possibly integrated into the scaffold and differentiated into chondrocytes [25].

Another unresolved question is the underlying biomechanics that enables cells to move into the scaffold. Both adult chondrocytes and chondroprogenitor cells are usually located within a proteoglycan-rich pericellular matrix of fibrillar collagen, so any theory concerning cell movement must explain how this obstacle to motility could be overcome. Two main ideas are discussed in the literature. One is based on the observations of Lee et al. [26], who showed that chondrocytes can interact with surrounding collagen fibres by bending them, and thus they could possibly move along these fibres. The other is based on the involvement of proteolytic activity in tissue invasion, as observed by Werb [27] and Mignatti et al. [28], which could enable chondrocytes to enzymatically digest and resynthesise their direct environment; such a process would not only allow movement, but could possibly account for synthesis of a COL2 matrix.

A potential bias concerning the presented successful treatment of this young patient represents the patient’s very young age. With regard to literature there is no information concerning the influence of biological or chronological age on healing response or scaffold transformation in the treatment of articular cartilage defects with cell-free scaffolds. Therefore additional information concerning larger patient collectives including different age groups is needed in order to recommend cell free scaffold as a general treatment procedure for articular cartilage defects.

## Conclusion

The case of this patient showed that cell-free COL1 scaffold could provide a suitable matrix allowing for cellular repopulation and synthesis of a COL2 structure in an articular chondral defect of the knee. To determine the origin of these cells and how exactly they move into the scaffold, further investigations are needed.

### Consent

Informed consent was obtained for taking the biopsy during arthroscopic treatment for the meniscal tear as well as for publishing the resulting data.

## Abbreviations

COL I: Type I collagen; COL II: Type II collagen.

## Competing interests

TE is consultant to Smith&Nephew, Endoscopy, Germany. The magnetic resonance imaging was supported by a research fund of Arthro Kinetics.

## Authors’ contribution

KFS mainly drafted the manuscript and aided in the follow-up examinations. JS provided critical revision and did aid in drafting of the manuscript. MBR was the leading radiologist and examined the magnetic resonance images. PR was the leading pathologist and provided the histological examinations. TE performed the surgery, carried out the follow-up examinations, and did aid in drafting and proofreading the manuscript. All authors read and approved the final manuscript.

## Pre-publication history

The pre-publication history for this paper can be accessed here:

http://www.biomedcentral.com/1471-2482/13/11/prepub
